# Quality of Life in Adults with Metabolic Dysfunction-Associated Fatty Liver Disease

**DOI:** 10.3390/ijerph182413145

**Published:** 2021-12-13

**Authors:** Tae-In Hwang, A-Lum Han

**Affiliations:** 1 Department of Family Medicine, Wonkwang University Hospital, Iksan 54538, Korea; hwangti123@gmail.com; 2 Institute of Wonkwang Medical Science, Wonkwang University College of Medicine, Iksan 54538, Korea

**Keywords:** metabolic dysfunction-associated fatty liver disease, population-based analysis, quality of life, stress

## Abstract

The aims of this study were as follows: to investigate the association between metabolic dysfunction-associated fatty liver disease (MAFLD) and health-related quality of life (HRQoL), to evaluate whether stress perception and mental health among patients with MAFLD affect HRQoL, and to identify the underrated burden on MAFLD patients. Nationwide data from the 5th Korean National Health and Nutrition Examination Survey (KNHANES V, 2010 to 2012) were used. MAFLD was defined by a fatty liver index (FLI) of ≥60, and the EuroQol-5D (EQ-5D) was used to assess HRQoL. Logistic regression analysis and odds ratios (ORs) were used to determine the associations of MAFLD with stress, mental health, and HRQoL. Previous suicidal impulse was not found to be significantly associated with HRQoL. The risk of MAFLD increased 1.265-fold with an increase in stress levels based on the stress perception rate (confidence index (CI): 1.046–1.530; *p* < 0.05), while it increased 1.091-fold with a 1-point decrease in the EQ-5D score (CI: 1.019–1.169; *p* < 0.05). HRQoL impairment and stress levels are associated with MAFLD. It is important to evaluate stress levels among MAFLD patients and implement stress management and HRQoL improvement strategies.

## 1. Introduction

Metabolic dysfunction-associated fatty liver disease (MAFLD) is a term that is globally emerging as a more appropriate nomenclature for non-alcoholic fatty liver disease (NAFLD) [1,2,3,4]. The term NAFLD has been used to describe excessive fat build-up in the liver with no other apparent cause, such as alcohol use [2]. This excessive fat deposition can induce hepatitis, progressing to non-alcoholic steatohepatitis (NASH) with complications, including liver cirrhosis, hepatic cancer, liver failure, or cardiovascular diseases. NAFLD is the most common cause of liver cirrhosis, accounting for 90% of idiopathic liver diseases. Moreover, it is also the most common liver disease worldwide [5]. Over the last two decades, the global prevalence of NAFLD has risen drastically in both sexes and all age groups, including adolescents [6,7]. It is prevalent in about 25% of the population worldwide and particularly very common in developed countries, such as Korea [8,9,10].

NAFLD has attracted significant attention from clinicians and scientists. Subsequently, both its pathophysiology and disease entity have come to be better understood. Despite these advances, however, the term NAFLD remains insufficient to demonstrate the multifactorial and heterogeneous nature of this disease [2,4]. Obesity, type-2 diabetes mellitus (DM), metabolic syndrome, and high fructose consumption all encourage the development of NAFLD. However, hepatic steatosis associated with metabolic diseases is hardly defined by a diagnosis of exclusion such as NAFLD. To address these concerns, in 2020 the Asian Pacific Association for the Study of the Liver selected the term MAFLD to replace and redefine NAFLD. Whereas NAFLD utilized exclusionary criteria, MAFLD takes advantage of various inclusionary diagnostic criteria. To define MAFLD, histological (biopsy), imaging, or blood biomarkers are first required to evidence fat accumulation within the liver (hepatic steatosis) [1,3]. The diagnostic criteria for MAFLD include obesity, type-2 DM, or the presence of two or more metabolic dysfunctions [1,3].

When diagnosing MAFLD, existing hepatic steatosis in adults can be diagnosed using blood biomarkers/scores alone when histological (biopsy) or imaging cannot be performed [11]. Biomarker/score-based diagnosis is suitable for extrapolating a large-scale epidemiological survey without requiring imaging modalities, such as abdomen ultrasound or computed tomography [11,12]. Among available scoring tools, the fatty liver index (FLI) meets the needs for investigating MAFLD owing to its cost-effectiveness, reliability, and handiness [13]. Recent research has described FLI as a simple and appropriate predictor of fatty liver diseases as well as a possible indicator of liver cirrhosis [14,15]. It has also been used in several studies on NAFLD in Korea [16,17,18]. Constitutive variables of FLI include triglycerides, body mass index (BMI), gamma-glutamyl transferase (GGT), and waist circumference (WC) [15]. Herein, FLI was used to diagnose MAFLD in Korean adults.

Stress is related to metabolic diseases and cardiovascular diseases [19]. Moreover, it often interplays with unhealthy behaviors, such as excessive alcohol consumption and smoking. The underlying pathophysiology is often expounded by an over-activated hypothalamic–pituitary–adrenal cortex (HPA) axis, stress-related hormone secretion, and inflammatory cytokine level increase [20].

Alterations in the composition of the gut flora alter host–microbiota interactions and lead to the dysregulation of the gut immune system, which is associated with the pathogenesis of several diseases, including type 2 DM, obesity, and MAFLD [21]. The gut microbiota primarily affects the host through immunological, metabolic-dependent, and metabolic-independent pathways [22]. The hypothalamus–pituitary–adrenal (HPA) axis, the body’s main neuroendocrine system which controls various metabolic processes in response to stress, interacts closely with the gut microbiota [22]. This close relationship between stress and chronic diseases may imply that chronic diseases are associated with a poor quality of life in patients [23,24]. However, studies on the quality of life and stresses of patients with MAFLD are still lacking, since most studies on MAFLD have focused on its pathophysiology and management. Further, existing studies on correlation with quality of life used the traditional NAFLD criteria [25].

To this end, we aimed to investigate the association between MAFLD and health-related quality of life (HRQoL), to evaluate whether stress perception and mental health among patients with MAFLD affect HRQoL and identify the underrated burden on MAFLD patients.

## 2. Materials and Methods

### 2.1. Study Design

In this study, we used the data of 17,476 survey participants aged 19 to 70 years old from the 5th Korean National Health and Nutrition Examination Survey (KNHANES V, 2010 to 2012) conducted by the Korean Centers for Disease Control and Prevention (KCDC). The exclusion criteria were as follows: missing required values (TG, GGT, WC, and BMI); viral hepatitis B and/or C (systematic history and/or blood tests); autoimmune hepatitis; drug-induced hepatitis; other liver diseases; and other medical conditions that may affect FLI levels [26].

KNHANES has been performed in Korea as a national wide survey every year since 1998. This annual survey comprises a health interview, a health check-up, and a nutritional survey to monitor the health and nutritional state of Koreans. The survey uses a stratified, multi-stage clustered probability sampling method. Each component is carried out by trained experts and clinicians.

KNHANES has obtained approval from the KCDC Agency Review Board. In accordance with the ethical standards of the Declaration of Helsinki, this study also obtained approval from the Wonkwang University Hospital’s clinical trial screening committee (IRB, approval number 2021-08-009) [26].

### 2.2. MAFLD Diagnosis

Imaging results and histologic work-up are virtually almost impossible to obtain from a nationwide large-scale survey such as KNHANES. Thus, we diagnosed hepatic steatosis in MAFLD using blood biomarker [27] scores. The presence of MAFLD was determined using a previously validated diagnostic index for fatty liver disease in Koreans. In a predictive model, FLI was used for diagnosing fatty liver diseases. Since Bedogni et al. modified the diagnostic criteria of FLI to improve its accuracy as a predictive index for fatty liver diseases in 2006, it has been widely used to diagnose fatty liver disease in Korea [14,15].

FLI was used for diagnosing MAFLD. The equation used was as follows:

FLI = (e(0.953 × ln(TG) + 0.139 × BMI + 0.718 × ln(GGT) + 0.053 × WC-15.745))/(1 + e(0.953 × ln(TG) + 0.139 × BMI + 0.718 × ln (GGT) + 0.053 × WC-15.745)) × 100

where TG, GGT, and WC denote triglyceride (mg/dL), γ-glutamyl transferase (U/L), and waist circumference (cm), respectively [14].

FLI ranged from 0 to 100. MAFLD was ruled out at a value of FLI < 30 and was confirmed if FLI ≥ 60. This yielded an area under the receiver operating characteristic of 0.85 (95%CI = 0.79–0.90) for diagnostic accuracy [14].

According to the European Association for the Study of the Liver guidelines, MAFLD was considered present and absent when FLI was ≥60 and <30, respectively [11].

For investigating diagnostic accuracy, subjects with an FLI value between 30 and 60 were excluded.

### 2.3. Anthropometric Measurements

Trained inspectors and examiners carried out all anthropometric and biochemical measurements. All parameters were recorded via individual measurements, personal observations, or clinical analyses. A stadiometer (Seca 225, Seca, Hamburg, Germany) and a mobile electronic weighing scale (GL-6000-20, G-tech, Seoul, Korea) were used to measure the heights (cm) and weights (kg) of the subjects in an examination gown, respectively. The resulting heights and weights were calculated to obtain BMI using the following BMI equation:

BMI = weight (kg)/square of height (m^2^)

WC was measured as recommended by the WHO guideline (the midline between the least palpable rib margin and the top of the iliac crest to the nearest 0.1 cm) [28]. Laboratory biochemical blood test samples were collected from each patient after 8 h of fasting. Collected blood samples were immediately transferred into plastic tubes, preserved in a refrigerator, and then transported to our central laboratory facility (Neodin Medical Institute, Seoul, Korea). Biochemical parameters, including fasting plasma glucose, hemoglobin A1c (HbA1c), aspartate aminotransferase, alanine aminotransferase, gamma-glutamyl transferase (ϓ-GT) level, and lipid profile (total cholesterol (TC), triglyceride (TG), high-density lipoprotein cholesterol (HDL-C), low-density lipoprotein cholesterol (LDL-C)) were obtained using an automatic chemistry analyzer (Hitachi 7600, Hitachi, Tokyo, Japan) [26].

### 2.4. Sociodemographic and Lifestyle Variables

The sociodemographic and lifestyle variables of the subjects were assessed using data on sex, smoking status, alcohol consumption, physical exercise, and educational level from KNHANES V.

With respect to smoking status, participants were classified as smokers if they had been smoking at the time of data collection. Alcohol consumption was assessed by measuring the amount of alcohol consumed per week in grams. Physical exercise was assessed using the International Physical Activity Questionnaire [27]. Regular exercise was defined as follows: physical exercise five times a week for more than 30 min each session or vigorous physical activity more than three times a week for more than 20 min per session. Education level was categorized into four stages: below elementary school graduation, middle school graduation, high school graduation, and above college graduation.

The degree of stress perception was assessed via the following answers.

1. I feel very much;
2. I feel a lot;
3. I feel a little;
4. I hardly feel any.

Items 1 or 2 and items 3 or 4 represent a high level of stress and a low level of stress, respectively.

A depressive episode was defined as experiencing a feeling of sadness or hopelessness for longer than two consecutive weeks interfering with daily life in the last year. Suicidal impulse was defined as experiencing any suicidal impulse in the last year. When the subject answered yes to that question, they were further asked about any suicide attempts. The EuroQol-5D (EQ-5D), a descriptive system introduced by EuroQol (Rotterdam, Netherlands) to evaluate the generic quality of life [29,30], was employed to investigate the health-related quality of life (HRQoL).

The preference-based HRQoL has been widely used in clinical trials and population studies in Korea. EQ-5D measures HRQoL using one question each for assessing five dimensions, including mobility, self-care, usual activities, pain/discomfort, and anxiety/depression. Answers can lead to the determination of 243 health states and may be converted into index scores anchored at 0 to 1 (death to perfect health) [30,31].

### 2.5. Statistical Analysis

All statistical analyses were performed using PASW statistics 23 (previously SPSS statistics) (SPSS version 23.0, IMP SPSS Inc., Chicago, IL, USA). In general, a complex sample plan for frequency analysis was used for performing the frequency analysis. Using a complex sample Rao–Scott-corrected chi-square test and a complex sample general linear model, statistics were processed to compare general characteristics as well as stress, suicide ideation, depression, HRQoL, and MAFLD.

A complex sample logistic regression procedure was used to investigate the correlation of MAFLD with stress, suicide ideation, depression, and HRQoL. A complex sample logistic regression analysis was performed and odds ratios (ORs) and 98% confidence intervals (CIs) were obtained by adjusting for the statistically significant variables to investigate the relationship between MAFLD and stress, suicide ideation, depression, and HRQoL. All data were provided as means ± standard errors (SEs) or percentages (%) for categorical variables. *p* values of < 0.05 were considered statistically significant.

## 3. Results

From the KNHANES V, 10,506 subjects (4250 male (49.9%) and 6207 females (50.1%)) who reliably completed the survey were selected for this study. A total of 4299 subjects satisfied the inclusion criteria of this study. In the MAFLD group, 75.6% of participants were male and 24.4% were female, with an average age of 45.29 ± 0.32 years. Of subjects with MAFLD, the level of education was elementary school graduation in 18.7%, middle school graduation in 13.5%, high school graduation in 35.5%, and more than college graduation in 32.4% (*p* < 0.05).

Table 1 summarizes the differences between the general characteristics, stress perception, mental health, and HRQoL among individuals with and without MAFLD.

Both rates of smoking and alcohol use were higher among individuals with MAFLD than among those without (41.4% vs. 23.1%, 73.3% vs. 56.6%; *p* < 0.0001). Compared to the MAFLD subjects, the non-MAFLD subjects showed higher values for high school graduation rate and college graduation rate. Compared to the non-MAFLD subjects, the MAFLD subjects showed higher values for all MAFLD-related variables, including age, WC, BMI, fasting glucose, HbA1c, TC, TG, and LDL-C, except the low HDL-C category (*p* < 0.05) (Table 1).

Table 2 delineates the association between MAFLD risk and general characteristics, stress, depression, suicide impulse, and HRQoL. A complex sample logistic regression test was used for analyzing the association between each MAFLD-related variable and MAFLD. The risk of MAFLD development was notably higher in males, middle school graduates, smokers, drinkers, and older individuals (OR 3.694 (3.184–4.285), OR 1.437 (1.157–1.784), OR 2.356 (2.015–2.754), OR 2.106 (1.800–2.465), OR 1.107 (1.066–1.150), respectively, *p* < 0.05). Every 1 unit in WC and BMI increased the risk of MAFLD (OR 1.304 (1.284–1.325), OR 1.832 (1.765–1.902), respectively, *p* < 0.05). Every 1 unit in FBS, HbA1c, TC, HDL-C, TC, and LDL-C enhanced the MAFLD risk (OR 1.023 (1.019–1.027), OR 1.534 (1.391–1.692), OR 1.015 (1.013–1.017), OR 0.938 (0.930–0.945), OR 1.015 (1.014–1.016), OR 1.007 (1.003–1.011), respectively, *p* < 0.05).

When EQ-5D decreased by 1, the risk of MAFLD increased (OR 1.077 (1.018–1.139), *p* < 0.05).

Table 3 delineates the association between MAFLD and stress, suicidal impulse, and HRQoL after adjustment for significant variables, including age, sex, level of education, tobacco use, and alcohol consumption. A complex sample logistic regression test was employed to analyze the relationship between MAFLD and stress perception, suicidal impulse, and HRQoL. The experience of a suicidal impulse showed no significant correlation with MAFLD occurrence. In subjects who answered the question regarding stress perception, the risk of MAFLD was higher (OR 1.265 (1.046–1.530) *p* < 0.05). When the EQ-5D score decreased by 1, the risk of MAFLD increased (OR 1.091 (1.019–1.169) *p* > 0.05).

## 4. Discussion

This study aimed to investigate the association between MAFLD, stress, mental health, and HRQoL among Korean adults. Stress and HRQoL showed an association with MAFLD. In this study, FLI was employed for MAFLD diagnosis due to its effectiveness and relevance for analyzing large-scale data.

A cross-sectional study conducted on Iranian adults reported the receiver operating characteristic curve to be 0.85 (95%CI = 0.79–0.90) for predicting hepatic steatosis in NAFLD [14]. Another study reported the diagnostic accuracy of FLI for steatosis to be more than 5% and yielded an AUROC of 0.97 (95% CI: 0.95–0.98). By excluding all subjects at the intermediate FLI score zone (60> and ≥30), the sensitivity and specificity of FLI were found to be 80.3% and 87.3%, respectively [13,15,32].

Few studies of FLI have been carried out using the newly revised definition of MAFLD in Asia since MAFLD was newly defined in 2020. One study tried to explore the optimal cut-off values of FLI for the diagnosis of steatosis in the liver in men and women using ultrasound as the reference standard in a large cross-sectional survey in China [33]. FLI accurately identified liver steatosis in the study. This study, conducted separately for men and women, presented each cutoff value of FLI for predicting liver steatosis in MAFLD [33].

Impaired HRQoL in NAFLD patients has been reported by numerous studies. Golani et al. performed a cross-sectional study on American adults (National Health and Nutrition Examination Survey 2001–2011) and reported that NAFLD patients were 18–20% more likely to report impaired HRQoL [24]. A metanalytic pragmatic literature review was performed by Kennedy-Martin et al. using five quantitative, two interventional, and one qualitative studies. Their study emphasized that patients with NASH experience various burdensome symptoms with a broad negative impact on HRQoL [34].

Another analytic study using data from three countries also revealed the association of NAFLD with HRQoL. That study pointed out the underrated burden on NAFLD patients, including the regular use of medical resources and symptoms of NAFLD [23]. Since hepatitis C virus (HCV) infection is associated with impaired HRQoL, it has often been compared with the HRQoL of NAFLD patients. Some studies have reported conflicting findings in terms of comparisons of HRQoL between NAFLD and HCV patients, specifically with regard to the degree of negative impact. Dan et al. concluded that NAFLD patients had significantly lower HRQoL than hepatitis B or C patients did. On the other hand, Golabi et al. reported that HRQoL scores were the lowest for HCV patients, followed by NAFLD patients. These differences may be attributed to the use of different HRQoL scoring indices (domain scores of the 29-item Chronic Liver Disease Questionnaire (CLDQ) in Dan’s study versus the NHANES HRQOL-4 questionnaire in Golabi’s study) [23,24,25].

To the best of our knowledge, no previous study has focused on HRQoL in MAFLD patients. Furthermore, this study was conducted in the Korean population, where there is an increasing MAFLD prevalence, while previous studies have mostly been conducted in Western countries.

In this study, HRQoL appeared to be lower in patients with MAFLD. After correction for relevant variables, it appeared that stress and HRQoL were associated with MAFLD.

Although further studies in this area are warranted, the promotion of the pathogenesis of MAFLD could potentially be explained by the following mechanisms.

Psychological problems, such as depression and stress, affect individuals by triggering physical responses, such as sympathetic tone upregulation, HPA axis activation, stress hormone secretion, and inflammatory cytokine release. Visceral fat accumulation caused by inflammatory cytokines and tumor necrotizing factor-alpha interrupts both insulin resistance and the leptin level [35]. As a result, this cascade of neural and molecular events can lead to the development of metabolic diseases [19,20,36,37,38,39]. These resulting metabolic dysfunctions further promote MAFLD. This hypothesis may suggest that individual stress susceptibility could be associated with MAFLD [19].

We speculated that the MAFLD had a negative impact on patients’ HRQoL due to the following reasons: (1) it leads to other metabolic and musculoskeletal diseases; (2) it incurs medical expenses and requires time; and (3) it is related to stress and mental health. Obesity, insulin resistance, and metabolic syndrome lead to the occurrence of MAFLD. The pathophysiologic mechanism of this disease also can affect further extra-hepatic diseases, such as type-II DM, CVD, CKD, and osteoarthritis. A previous study reported that knee OA was 6.331 times higher in terms of prevalence in patients with MAFLD [40]. Furthermore, a retrospective study from MarketScan Commercial claims (2006–2016) reported that the annual medical expenditure of NAFLD patients without advanced liver diseases was estimated at $23,860 in the USA [41]. A previous study reported that lifetime rates of major depressive disorder were significantly increased in this group (OR: 3.8; 95% CI: 1.7–14.9; *p* = 0.005) [39]. The stress perception rate was found to be higher in NAFLD patients in a previous study. Additionally, 24 h urinary free cortisol excretion and the serum cortisol level after dexamethasone challenge were also found to be higher in NAFLD patients. These results also corresponded to the severity of liver histology findings [42].

A previous study using the Nonalcoholic Steatohepatitis Clinical Research Network and Short Form 36 survey reported a significantly lower HRQoL in NAFLD patients with NASH and cirrhosis [14].

Another study using the domain scores of the 29-item CLDQ and EQ-5D reported that NAFLD patients showed significantly lower quality of life scores than HBV and HCV patients [23]. The EQ-5D scoring system was also used in this study.

The EQ-5D index has one question for each of the five dimensions, which concern mobility, self-care, usual activities, pain/discomfort, and anxiety/depression. The answers can be converted into index scores anchored at 0 to 1 (0 for death, 1 for perfect health). Since the validity of the quality of life questionnaire of EQ-5D for Koreans was investigated by Nam et al., the EQ-5D has been extensively used in Korea [29,30,31].

Although not an important point of contention in this study, we did find significant differences in educational attainment between MAFLD and non-MAFLD subjects. Compared to MAFLD subjects, non-MAFLD subjects showed higher values for the high school graduation rate and university graduation rate. The reason for this could not be determined in our analysis, but as some studies have noted, it may be that the more educated group are better at self-management [16].

After correction for related variables such as age, sex, level of education, tobacco use, and alcohol consumption, the risk of MAFLD was found to increase 1.265-fold with an increase in stress levels based on the stress perception rate, while it increased 1.091-fold with a 1-point decrease in the EQ-5D score. These results show that both stress and HRQoL in patients should be considered for proper management.

This study had some limitations. Firstly, because this study analyzed cross-sectional data, the causal relationship between stress, HRQoL, and MAFLD was only explained in a limited manner. Secondly, despite adjustment, a diagnosis of MAFLD based on blood scoring is still less accurate than a diagnosis based on liver biopsy and abdominal ultrasound. Thirdly, some other variables that potentially influence MAFLD were not dealt with in this paper.

Despite these weaknesses, this study had the following strengths. Firstly, this study delineated the significance of psychological aspects via explicating the association between stress and MAFLD. More importantly, this study elucidates that HRQoL was associated with MAFLD among Korean adults based on the newly revised nomenclature.

These results provided an understanding of the complex pathophysiology of this disease. This paper also proposed a pivotal strategy for establishing proper management through a multi-faceted approach to MAFLD care.

## 5. Conclusions

HRQoL impairment and stress levels were found to be associated with MAFLD. After adjustment for important variables including age, gender, education level, tobacco use, and alcohol consumption, associations between MAFLD and stress were identified. The risk of MAFLD increased by 1.265 times as the stress perception rate increased. Similarly, an association between MAFLD and HRQoL impairment was also confirmed, with it increasing 1.091-fold as HRQoL impairment increased. It is important to evaluate stress levels among MAFLD patients and implement stress management and HRQoL improvement strategies.

## Figures and Tables

**Table 1:** Differences between general characteristics and mental health, stress, HRQoL, and MAFLD.

		Total Subjects (10,506)	MAFLD = No (9155)	MAFLD = Yes (= 1351)	*p*
Sex	male	4299 (49.9)	3396 (45.7)	903 (75.6)	<0.0001
	female	6207 (50.1)	5759 (54.3)	448 (24.4)	
Education level	Elementary	2649 (18)	2284 (17.9)	365 (18.7)	0.004
	Middle	1163 (10.3)	967 (9.7)	196 (13.5)	
	High	3425 (38.2)	3019 (38.6)	406 (35.5)	
	College	3116 (33.5)	2746 (33.7)	370 (32.4)	
Smoking		1971 (25.7)	1529 (23.1)	442 (41.4)	<0.0001
Exercise		1021 (9.9)	878 (9.9)	143 (9.9)	0.997
Alcohol		5378 (58.9)	4484 (56.6)	894 (73.3)	<0.0001
Age		45.29 ± 0.32	44.46 ± 0.32	46.98 ± 0.5	<0.0001
WC		81.01 ± 0.16	78.74 ± 0.14	94.9 ± 0.29	<0.0001
BMI		23.62 ± 0.05	22.89 ± 0.04	28.22 ± 0.12	<0.0001
Fasting glucose		96.27 ± 0.29	94.25 ± 0.28	108.61 ± 0.95	<0.0001
HbA1c		5.8 ± 0.02	5.71 ± 0.02	6.25 ± 0.05	<0.0001
TC		187.82 ± 0.51	184.89 ± 0.51	205.7 ± 1.25	<0.0001
HDL-C		49.53 ± 0.18	50.56 ± 0.19	43.24 ± 0.33	<0.0001
TG		132.14 ± 1.46	110.3 ± 0.88	265.26 ± 6.3	<0.0001
LDL-C		111.96 ± 0.7	110.86 ± 0.72	118.5 ± 1.98	<0.0001
Stress perception		0.28 ± 0.01	0.27 ± 0.01	0.3 ± 0.02	0.073
Suicidal impulse		0.14 ± 0.004	0.14 ± 0.005	0.15 ± 0.01	<0.0001
Depression		0.13 ± 0.004	0.13 ± 0.004	0.12 ± 0.01	0.894
EQ-5D		0.95 ± 0.001	0.95 ± 0.001	0.94 ± 0.004	0.018

Abbreviations: MAFLD: metabolic dysfunction-associated fatty liver disease; HRQoL: health-related quality of life; EQ-5D, EuroQol-5 Dimension; WC: waist circumference; BMI, body-mass index; HbA1c, hemoglobin A1c; TG, triglyceride; LDL-C, low-density lipoprotein cholesterol; TC, total cholesterol; HDL-C, high-density lipoprotein cholesterol. Definitions: smoking: the percentage of individuals who currently smoke and have smoked at least 100 cigarettes (5 packs) in their life time; alcohol: the percentage of individual with current alcohol use; FLI, fatty liver index = FLI = (e(0.953 × ln(TG) + 0.139 × BMI + 0.718 × ln(GGT) + 0.053 × WC-15.745))/(1 + e(0.953 × ln(TG) + 0.139 × BMI + 0.718 × ln (GGT) + 0.053 × WC-15.745)) × 100; MAFLD, FLI ≥ (GGT) + 0.053 × WC- < 30; stress: the percentage of stress experienced during everyday life. Values were presented as a number (%) or as a mean ± standard deviation. The *p*-value was determined through the complex sample Rao–Scott-adjusted chi-square test and complex sample-generalized linear model T test.

**Table 2:** General characteristics and the association between MAFLD and stress, mental health, and HRQoL.

		OR	(95% CI)	*p*
Sex	Male	3.694	(3.184–4.285)	<0.0001
	Female	1	Reference	
Education level	Elementary	1.087	(0.908–1.303)	0.363
	Middle	1.437	(1.157–1.784)	0.001
	High	0.955	(0.791–1.152)	0.628
	College	1	Reference	
Smoking		2.356	(2.015–2.754)	<0.0001
Exercise		1.000	(0.786–1.273)	0.997
Alcohol		2.106	(1.800–2.465)	<0.0001
Age		1.107	(1.066–1.150)	<0.0001
WC		1.304	(1.284–1.325)	<0.0001
BMI		1.832	(1.765–1.902)	<0.0001
Fasting glucose		1.023	(1.019–1.027)	<0.0001
HbA1c		1.534	(1.391–1.692)	<0.0001
TC		1.015	(1.013–1.017)	<0.0001
HDL-C		0.938	(0.930–0.945)	<0.0001
TG		1.015	(1.014–1.016)	<0.0001
LDL–C		1.007	(1.003–1.011)	<0.0001
Stress perception		1.170	(0.989–1.384)	0.067
Suicidal impulse		1.101	(0.901–1.346)	0.346
Depression		0.985	(0.792–1.226)	0.894
EQ–5D	−0.1 (realignment parameter)	1.077	(1.018–1.139)	0.010

Abbreviations: MAFLD: metabolic dysfunction-associated fatty liver disease; HRQoL: health-related quality of life; EQ-5D, EuroQol-5 Dimension; WC: waist circumference; BMI, body-mass index; HbA1c, hemoglobin A1c; TG, triglyceride; LDL-C, low-density lipoprotein cholesterol; TC, total cholesterol; HDL-C, high density lipoprotein cholesterol. Definitions: smoking: the percentage of individuals who currently smoke and have smoked at least 100 cigarettes (5 packs) in their life time; alcohol: the percentage of individual with current alcohol use; FLI, fatty liver index = FLI = (e(0.953 × ln(TG) + 0.139 × BMI + 0.718 × ln(GGT) + 0.053 × WC-15.745))/(1 + e(0.953 × ln(TG) + 0.139 × BMI + 0.718 × ln (GGT) + 0.053 × WC-15.745)) × 100; MAFLD, FLI ≥ (GGT)on-MAFLD, FLI < 30; stress, percentage of stress during everyday life. The *p*-value was determined through the complex sample logistic regression test.

**Table 3:** Association between MAFLD and stress, mental health, and HRQoL.

	OR	(95% CI)	*p*
Stress	1.265	(1.046–1.530)	0.016
Suicidal impulse	1.109	(0.893–1.377)	0.347
EQ-5D (−0.1)	1.091	(1.019–1.169)	0.013

Abbreviations: MAFLD: metabolic dysfunction-associated fatty liver disease; HRQoL: health-related quality of life; EQ-5D, EuroQol-5 Dimension. Definitions: stress, the percentage of stress during everyday life; suicidal impulse, the experience of suicidal impulse in the past year; depression, the experience of a feeling of sadness or hopelessness for longer than two consecutive weeks interfering with daily life in the past year. Adjusted for sex, age education level, smoking, and alcohol. ORs and 95% CI were determined through the complex sample logistic regression test.

## Data Availability

Publicly available datasets were analyzed in this study, which could be used after obtaining approval from the relevant authorities. This data can be found here https://knhanes.kdca.go.kr/knhanes/sub03/sub03_02_05.do (accessed on 17 October 2021).
